# Advanced Preparation and Application of Cellulose

**DOI:** 10.3390/polym18091099

**Published:** 2026-04-30

**Authors:** Marta Fernandes, Jorge Padrão

**Affiliations:** Centre for Textile Science and Technology (2C2T), University of Minho, Campus de Azurém, 4800-058 Guimarães, Portugal; padraoj@2c2t.uminho.pt

Growing environmental awareness of global warming, energy crises, and waste generation is driving demand for renewable low-impact products. The scientific community is increasingly focused on developing sustainable and renewable materials for future applications, with particular interest in nanocellulose. Cellulose is a biobased polymer that stands out as one of the most prominent candidates in this area, due to its abundant availability from various sources.

Wood pulp is the main source for industrial cellulose production; however, slow tree growth drives interest in annual fast-growing plants. Sumarago et al. studied the effects of acid concentration and temperature on the yield and properties of nanocellulose extracted from Maguey fiber. A nanocellulose yield of 81.58% (50 °C and 50 wt.% H_2_SO_4_) with high crystallinity, good thermal stability, and strong colloidal stability corroborates this fiber as a sustainable and effective raw material for nanocellulose production [1]. The packaging industry uses a considerable amount of recycled cellulose pulp. Klemenčić et al. investigated the removal of heavy metals during paper and cardboard recycling from pharmaceutical packaging via deinking flotation. The results gathered indicate that the presence of adhesives and metal electronegativity affect separation. Nevertheless, the recycling process effectively removes certain heavy metals, indicating strong potential for safe cardboard recycling [2].

Cellulose can be chemically modified through its abundant and highly reactive hydroxyl groups, enabling tailored functionalities. However, strong hydrogen bonding makes it insoluble in common organic solvents and water. Thus, homogeneous modification processes are increasingly explored to improve cellulose modification efficiency and homogeneous application. Yoshizawa et al. developed aromatic oxy-ionic liquids (ILs) for the modification of cellulose, achieving fast (30 min) and efficient esterification with a high degree of substitution and uniform structures, overcoming the side effects of acetyl groups common in carboxylate ILs [3]. Unlike cellulose, industrially synthesized cellulose ethers are often soluble in water, where they form rod-like conformations in solution. Yoshida et al. investigated the conformation of hydroxyethyl cellulose ether in aqueous solutions, concluding that the molecules behave like rigid rod-like particles across a wide molar-mass range, regardless of molar mass [4]. Ogiwara et al. investigated the cholesteric liquid crystals derived from hydroxypropyl cellulose esterified with two alkanol side-chain groups to control the helical structure and optical properties. These materials have tunable color responses to temperature, highlighting their potential for sustainable photonic and optical applications [5]. In the work carried out by Perrin et al., using a non-chemical approach, it was shown that low-frequency ultrasound treatment affects cellulose nanocrystals by breaking aggregates and reducing particle size. The treatment improves the rheology of suspensions and promotes liquid-crystal formation, while leaving chemical structure, crystallinity, surface charge, and hydrophilicity unchanged, enhancing their use in stabilizing Pickering emulsions [6].

In energy applications, cellulose is an alternative material for replacing non-renewable sources, satisfying the currently high demand for sustainability in this fast-growing sector. It has attractive properties such as high carbon content, ease of surface modification and doping, three-dimensional structure, high specific surface area, and high thermal and structural stability. Villarreal-Rueda et al. investigated the production of activated carbon from bacterial nanocellulose grown in fique juice for use in supercapacitor electrodes. These materials, chemically activated with KOH at 800 °C, created porous carbons, achieving a high surface area (up to 893 m^2^/g) and exhibiting excellent electrochemical performance for sustainable energy storage [7]. Kroff et al. evaluated the use of nanocellulose as a reducing agent and carbon source in the microwave-assisted hydrothermal synthesis of lithium iron phosphate/carbon cathodes [8]. Puente-Córdova et al. highlighted the potential of ethyl cellulose for electronic and dielectric applications by analyzing the electrical conduction mechanisms under alternating and direct current electric fields, where they concluded that electric conduction is governed by the mobility of the chain segments [9].

Addressing water pollution is a critical environmental challenge. The intrinsic hydrophilicity and recycling challenges of cellulose-based aerogels have hindered their practical application as sorbents for oil and organic pollutant cleaning. To overcome this, Zhai and Yuan fabricated a cellulose-based aerogel incorporating Fe_3_O_4_ nanoparticles via a simple freeze-drying method, followed by chemical vapor deposition, resulting in superhydrophobic, magnetically recoverable and reusable composites for treating oil pollution in water [10]. In the work of Pérez-Silva et al., cellulose acetate capsules functionalized with Cyanex 923 for removing cadmium ions from aqueous solutions and battery leachates were explored [11].

Cellulose is widely utilized in biomedical engineering due to its excellent biocompatibility, tunable properties, and renewable nature. For example, Merivaara et al. developed lyophilized nanofibrillated cellulose hydrogels with tunable stiffness for three-dimensional cell cultures. The hydrogels have demonstrated success in forming viable 3D spheroids of mesenchymal stem cells and cancer cells, offering an efficient alternative for drug screening [12]. In another work, Chelades et al. investigated ethyl cellulose-ethanol gel as a controlled alternative to liquid ethanol for percutaneous ablation. Experiments in ex vivo swine liver established relationships between infusion volume, concentration, and needle gauge to predict the size of the ablation zone, aiming to optimize injection protocols for controlled, minimally invasive tumor ablation therapies [13].

The versatility of cellulose is unquestionable, and further promotes its relevance in adding an important sustainability factor to advanced critical applications.
